# Named Entity Recognition for Chinese Cancer Electronic Health Records—Development and Evaluation of a Domain-Specific BERT Model: Quantitative Study

**DOI:** 10.2196/76912

**Published:** 2025-11-14

**Authors:** Junbai Chen, Butian Zhao, Xiaohan Tian, Zhengkai Zou, Ruojia Wang, Jiarui Wu, Songxing Du, Fengying Guo

**Affiliations:** 1 School of Management, Beijing University of Chinese Medicine, No. 11, North Third Ring Road East, Chaoyang District, Beijing, 100029, China, 86 13811833948; 2 Department of Information Management, Peking University, Beijing, China; 3 Information Center, Dongfang Hospital, Beijing, China

**Keywords:** BERT, named entity recognition, cancer, electronic health records, deep learning

## Abstract

**Background:**

The unstructured data of Chinese cancer electronic health records (EHRs) contains valuable medical expertise. Accurate medical entity recognition is crucial for building a medical-assisted decision system. Named entity recognition (NER) in cancer EHRs typically uses general models designed for English medical records. There is a lack of specialized handling for cancer-specific records and limited application to Chinese medical records.

**Objective:**

This study aims to propose a specific NER model to enhance the recognition of medical entities in Chinese cancer EHRs.

**Methods:**

Desensitized inpatient EHRs related to breast cancer were collected from a leading hospital in Beijing. Building upon the MC Bidirectional Encoder Representations from Transformers (BERT) foundation, the study further incorporated a Chinese cancer corpus for pretraining, resulting in the construction of the ChCancerBERT pretrained model. In conjunction with dilated-gated convolutional neural networks, bidirectional long short-term memory, multihead attention mechanism, and a conditional random field, this model forms a multimodel, multilevel integrated NER approach.

**Results:**

This approach effectively extracts medical entity features related to symptoms, signs, tests, treatments, and time in Chinese breast cancer EHRs. The entity recognition performance of the proposed model surpasses that of the baseline model and other models compared in the experiment. The *F*_1_-score reached 86.93%, precision reached 87.24%, and recall reached 86.61%. The model introduced in this study demonstrates exceptional performance on the CCKS2019 dataset, attaining a precision rate of 87.26%, a recall rate of 87.27%, and an impressive *F*_1_-score of 87.26%, surpassing that of existing models.

**Conclusions:**

The experiments demonstrate that the approach proposed in this study exhibits excellent performance in NER within breast cancer EHRs. This advancement will further contribute to clinical decision support for cancer treatment and research. In addition, the study reveals that incorporating domain-specific corpora in clinical NER tasks can further enhance the performance of BERT models in specialized domains.

## Introduction

### Background

Cancer is one of the leading causes of mortality [1], imposing a significant psychological burden on patients and potentially triggering mental health disorders such as anxiety and depression [2]. The proliferation of electronic health records (EHRs) has provided a crucial source of demographic information, medical history, diagnostic tests, and clinical treatment data for cancer research, aiding in better diagnosis, prognosis, and treatment of the disease [3]. However, the large amount of unstructured text in EHRs presents significant challenges for clinical research and analysis. Structuring this unstructured information is essential for subsequent data analysis and mining to support clinical decision-making. Named entity recognition (NER) is a critical component of natural language processing (NLP), plays a key role in this process by identifying and classifying entities, allowing for transforming raw clinical texts into structured data, thereby enabling large-scale data mining and supporting intelligent health care applications [4].

In recent years, many studies have explored different NER methods to structure EHRs. The widespread application of pretrained models such as Bidirectional Encoder Representations from Transformers (BERT) has significantly enhanced NER performance in the medical field [5,6]. These improvements are especially valuable in the medical domain, where precise recognition of specialized entities directly impacts the reliability of downstream analyses including cohort selection, clinical decision support, and the construction of medical knowledge bases [7,8]. However, most of these studies focus on English texts, and the differences between English and Chinese in terms of language expression may limit the generalizability of English medical NER methods when directly applied to Chinese medical texts.

Compared to medical texts in common diseases, cancer EHRs contain richer and more complex information. These records span multiple fields, including medicine, biology, and pharmacology, and encompass specific medical terminologies such as cancer staging, treatment plans, and drug names. They also include diagnostic and treatment records like surgical notes and pathology results, with a prevalent occurrence of nested entities. In current NER tasks for the cancer domain, models such as BERT and other deep learning approaches based on general corpora are often used without fine-tuning for the numerous specialized terms and specific expressions in this field, which may result in errors or omissions. In addition, there is no publicly available dataset benchmark for the cancer domain. Therefore, NER tasks for Chinese cancer EHRs remain both challenging and necessary.

To address the aforementioned issues, this study contributes the following:

1. We retrained the MC-BERT model using a corpus specific to the Chinese cancer domain, resulting in the ChCancerBERT pretrained model tailored for NER tasks in the Chinese cancer field. This approach effectively enhances the semantic representation capabilities of pretrained models in a specialized domain.
2. In the Chinese medical domain, we built a hybrid model to capture temporal features and bidirectional semantic information. This combination enhances the model’s ability to perform multidimensional feature modeling.
3. To validate the model’s effectiveness, we created a Chinese breast cancer EHR corpus with manual annotations. We applied various models to this corpus, and the experimental results demonstrated that our proposed model outperforms existing models, achieving superior performance. We also applied the model proposed in this study to the CCKS2019 dataset and compared the results with those from existing studies for evaluation.

### Related Work

In the medical field, prior research has developed various NER methods for extracting information from unstructured data such as EHRs. Table 1 summarizes related studies and methods for clinical NER in the medical field.

NER techniques can be categorized into 3 main types: dictionary and rule-based methods, machine learning–based methods, and deep learning–based methods. Dictionary and rule-based NER methods rely primarily on researchers analyzing textual writing rules and establishing dictionaries, then using regular expression matching to achieve entity recognition and information extraction. For instance, Najafabadipour et al [9] extracted multiple cancer concepts such as tumor staging, mutation status, and patient presentation from lung cancer clinical records using regular expressions and the UMLS8 dictionary. Yim et al [10] used rule-based extraction to identify 3 liver cancer entities: tumor size, staging level, and percentage of tumors invading the liver. These methods are straightforward and efficient but lack generalizability and require significant human effort to build dictionaries [10]. Machine learning–based methods use sequence labeling and train models such as hidden Markov model, conditional random fields (CRFs), and maximum entropy Markov model on large manually annotated feature corpora to achieve NER. For example, Savova et al [11] developed the DeepPhe software, which uses a combination of rules, domain knowledge bases, and machine learning to extract cancer phenotypes from clinical records. Weegar et al [12] used a CRF model to extract cervical cancer symptom information.

In recent years, deep learning methods have made significant progress in medical NER tasks, demonstrating substantial advantages in NLP compared to traditional feature-based machine learning methods. These approaches typically predict the boundaries and types of entities by labeling each word, thereby capturing deeper and more abstract features. For instance, An et al [13] used a bidirectional long short-term memory (BiLSTM)-CRF model with a multihead attention (MHA) mechanism to perform Chinese clinical EHR NER. Kong et al [14] combined multilayer convolutional neural networks (CNNs) with an attention mechanism to improve NER in Chinese EHRs. The emergence of pretrained models like BERT has further enhanced the performance of NER. In the medical domain, existing research shows that BERT-based models can be effectively applied to medical information extraction tasks. Li et al [5] achieved excellent results using a BERT-BiLSTM-CRF model on the CCKS2018 and CCKS2019 datasets. Li et al [15] proposed an A Lite Bidirectional Encoder Representations from Transformers (ALBERT)-based model with a MHA mechanism for Chinese medical NER, validated on the CCKS2019 dataset. Chen et al [6] constructed a hybrid model combining MC-BERT, BiLSTM, CNN, MHA, and CRF to achieve NER in Chinese EHRs. Most of these studies primarily applied their deep learning models to publicly available datasets such as CCKS2017 and CCKS2019, without further testing them on specific medical departments or diseases.

Existing research indicates that using domain-specific text as training data, as opposed to general language models, can yield better performance. For the cancer field, Zhou et al [7] proposed CancerBERT, a pretrained model specifically designed for the English language in the cancer domain. Currently, there is no similar model for the Chinese cancer domain. Given the differences between Chinese and English in terms of vocabulary, sentence structure, grammatical rules, and semantic expression, directly applying models trained on English corpora may lead to suboptimal results.

**Table 1.** Summary of related work on clinical named entity recognition methods in the medical and cancer domains.

Proposal	Approach	Method	Domain	Domain model	Benchmark	Language
Najabadipour et al 2018 [9]	Rules	Rule-based methods	Cancer	Yes	No	Spanish
Yim et al.2016 [10]	Rules	Rule-based methods	Cancer	Yes	No	English
Savova et al 2017 [11]	Rules and machine learning	Rule-based methods	Cancer	Yes	No	English
Weegar et al 2015 [12]	Machine learning	CRF^a^	Cancer	No	No	English
An et al 2022 [13]	Deep learning	BiLSTM^b^-MHA^c^-CRF	Medical	No	Yes	Chinese
Kong et al [14]	Deep learning	CNN^d^	Medical	No	Yes	Chinese
Li et al 2022 [5]	Deep learning	ALBERT^e^-IDCNN^f^-MHA-CRF	Medical	No	Yes	Chinese
Li et al 2020 [5]	Deep learning	BERT^g^-BiLSTM-CRF	Medical	No	Yes	Chinese
Chen et al 2022 [6]	Deep learning	MC-BERT-BiLSTM-CNN-MHA-CRF	Medical	Yes	Yes	Chinese
Li et al 2023 [16]	Deep learning	MC-BERT GCN^h^-CRF	Medical	Yes	Yes	Chinese
Zhou et al 2022,2023 [7,8]	Deep learning	CancerBERT	Cancer	Yes	No	English

a CRF: conditional random field.

b BiLSTM: bidirectional long short-term memory.

c MHA: multihead attention

d CNN: convolutional neural network.

e ALBERT: A Lite Bidirectional Encoder Representations from Transformers.

f IDCNN: iterated dilated convolutional neural network.

g BERT: Bidirectional Encoder Representations from Transformers.

h GCN: graph convolutional network.

## Methods

### Ethical Considerations

Based on Article 32 of the *Measures for Ethical Review of Life Sciences and Medical Research Involving Human Subjects* [17] research using human data or biological samples that does not cause harm to the human body, does not involve sensitive personal information or commercial interests, is eligible for exemption from ethical review. This exemption is intended to reduce unnecessary burdens on researchers and to facilitate the progress of life sciences and medical research. In our study, the dataset used has undergone thorough anonymization, with all personal patient information removed. In addition, the patients involved in the clinical activities at the hospital have already signed the relevant informed consent forms, confirming their agreement to the use of their data for research purposes. Therefore, as per the national regulations, our study did not require ethical review. The data were granted permission to be accessed by SD, who is the director of the Information Center of Dongfang Hospital Beijing University of Chinese Medicine, responsible for managing all the hospital's data, and he is listed as a contributing author.

### Model Construction

**Figure 1.**
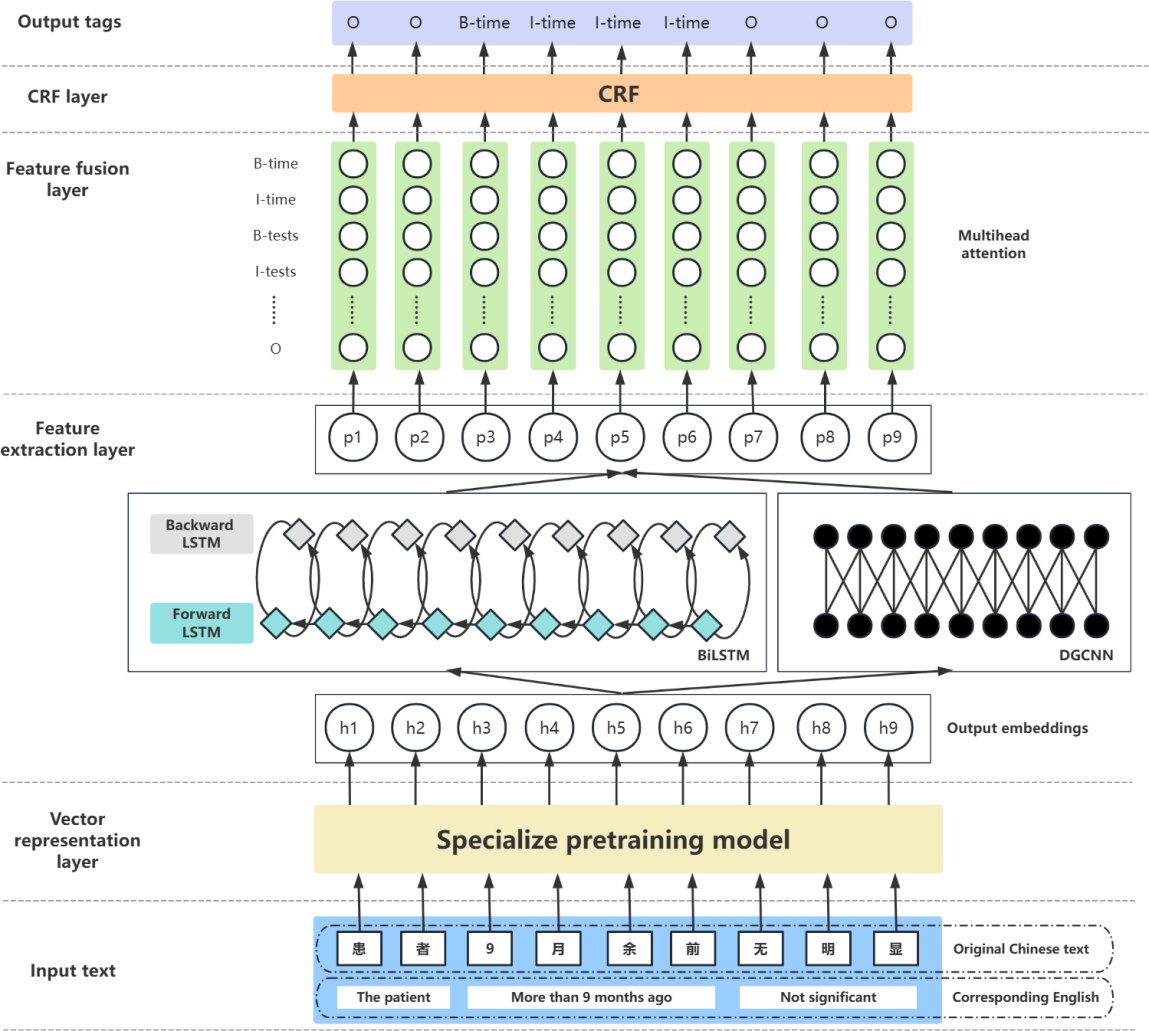
The architecture of the proposed model, for ease of viewing and understanding, the input text examples in the figure are accompanied by corresponding English translations. BiLSTM: bidirectional long short-term memory; CRF: conditional random field; DGCNN: dilated-gated convolutional neural network; LSTM: long short-term memory.

The proposed model architecture in this study is illustrated in Figure 1.

The model comprises 4 parts: the vector representation layer, feature extraction layer, feature fusion layer, and CRF layer. The Chinese cancer EHR texts are input into the model, where the input text is segmented into individual characters to form the basic sequence $X=\{x_{1},x_{2},\ldots ,x_{i}\}$, where $x_{i}$ is the *i*-th character. Initially, in the vector representation layer, the input sequences $X=\{x_{1},x_{2},\ldots ,x_{i}\}$ are represented as semantically rich character-level embedding vectors $h_{i}$ using the autonomously trained BERT model tailored to the Chinese cancer domain (ChCancerBERT), forming the embedding sequence $H=\{h_{1},h,\ldots ,h_{i}\}$. Subsequently, the sequence $H=\{h_{1},h,\ldots ,h_{i}\}$ is fed into the BiLSTM and dilated-gated convolutional neural network (DGCNN) models within the feature extraction layer. The feature extraction layer uses BiLSTM to extract long-distance dependency associations and temporal features from the sequences, while DGCNN captures spatial features. In the feature fusion layer, a MHA mechanism with enhanced generalization capability dynamically fuses the features extracted by BiLSTM and DGCNN to enhance the model’s feature representation capability. The CRF layer imposes constraints on the output to obtain the optimal labeling results. Finally, the model outputs a set of label sequences $Y=\{y_{1},y,\ldots ,y_{i}\}$, where each label $y_{i}$ corresponds to a character in the input text. For instance, “B-TIME” denotes the beginning of a TIME entity, while “I-TIME” denotes the inside of a TIME entity, with the label sequence indicating the model’s classification results for each character.

### Chinese Cancer Specialized BERT

**Figure 2.**
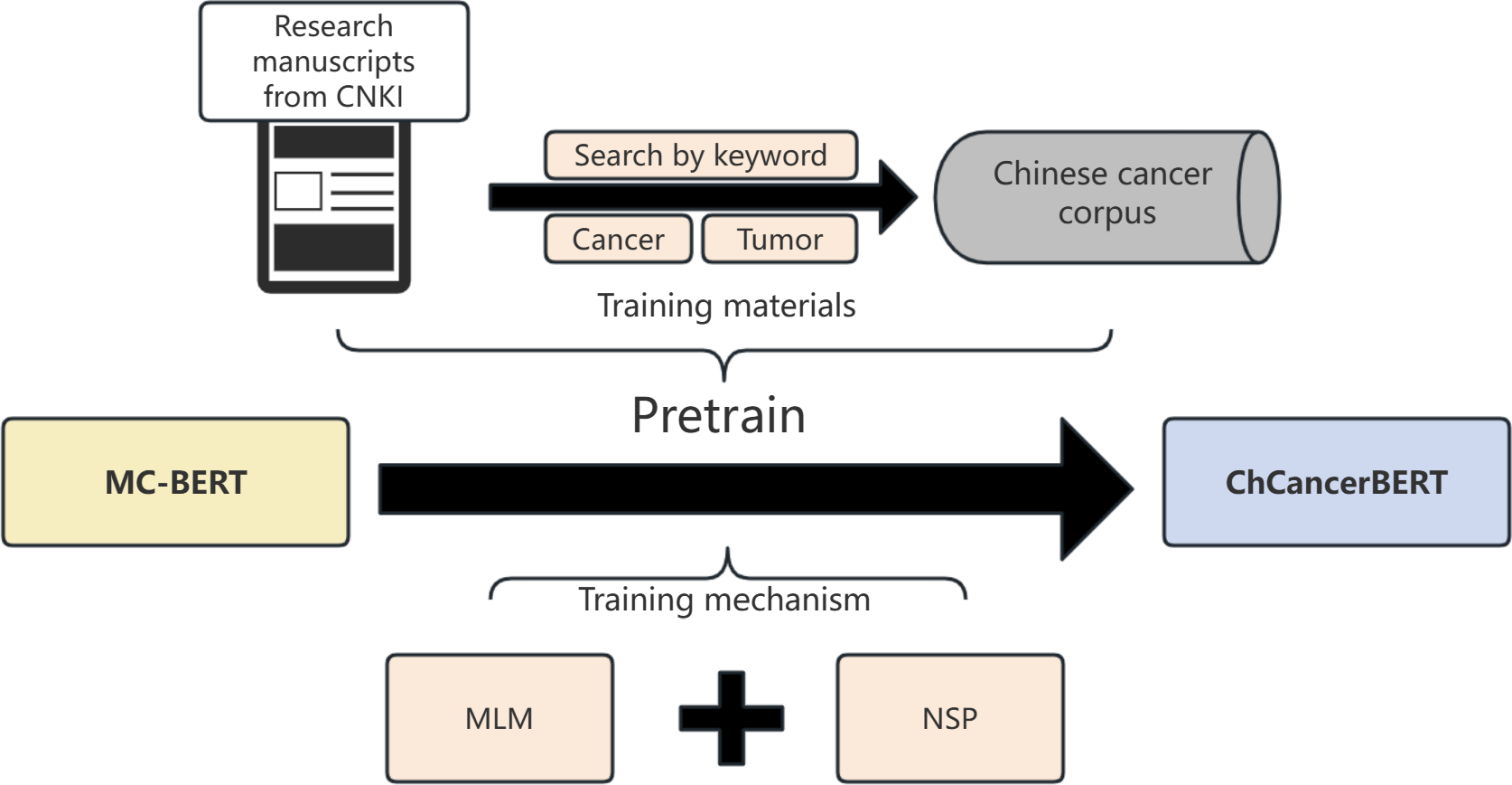
The training process of ChCancerBERT models. BERT: Bidirectional Encoder Representations from Transformers; CNKI: China National Knowledge Infrastructure; MC: ;MLM: masked language model; NSP: next sentence prediction

To better capture semantics in cancer electronic medical records, this study trained a specialized BERT model tailored for the Chinese cancer domain (ChCancerBERT) to enhance the performance of extracting cancer-related phenotypes. Figure 2 illustrates the training process of the ChCancerBERT model.

Pretrained models are typically based on large-scale general corpora for data pre-training. Depending on the specific needs, they can also incorporate domain-specific vocabulary to calculate the probability P(S) of all possible language sequences.


(1) $$P\left(S\right)=p\left(\omega_{1},\omega_{2},\ldots ,\omega_{n}\right)=\prod_{i=1}^{n}p\left(\omega |\omega_{1},\omega_{2},\ldots ,\omega_{i-1}\right)$$

The most commonly used pretrained model is the BERT model, a deep bidirectional language representation model that offers significant advantages in capturing textual semantic information and entity extraction [18-20]. Although BERT achieves good results in most domains, its generic nature may limit its ability to fully capture the specialized terminology and semantic nuances of the medical field [15]. MC-BERT fine-tuned on BERT by using different pretraining strategies to incorporate medical-specific and related vocabulary, enabling superior performance in medical NER tasks [6].

The ChCancerBERT model proposed in this study is further pretrained based on the MC-BERT model. The specific steps are as follows: firstly, a Chinese cancer corpus was constructed. Using keywords “cancer” and “tumor,” a search strategy retrieved relevant Chinese-language literature from the China National Knowledge Infrastructure (CNKI) database. The corpus included abstracts from the first 6000 articles, organized chronologically, resulting in a comprehensive dataset of 3,268,570 tokens. This dataset ensures that ChCancerBERT is exposed to terminology and context unique to the cancer domain, thus improving its capacity for semantic understanding. Second, the pretraining of ChCancerBERT used both masked language model and next sentence prediction tasks to enhance semantic representation. Based on the masked language model, the approach involves randomly masking certain characters within a sentence to learn the semantic information of each character in context. This encourages ChCancerBERT to learn nuanced semantic relationships, particularly for specialized terminology within cancer-specific contexts. In addition, the next sentence prediction model is used to learn the association between 2 consecutive sentences, enabling it to capture long-distance dependencies and contextual flows frequently present in medical records. This step is particularly beneficial for understanding the multisentence descriptions often found in medical documents.

### BiLSTM Model

Long short-term memory (LSTM) is a specialized form of recurrent neural network that can capture larger and longer-distance information. By using a set of gate controllers, LSTM effectively addresses the gradient vanishing and exploding problems inherent in traditional recurrent neural networks [21]. An LSTM, the input gate $i_{t}$ controls information into the current cell, the forget gate $f_{t}$ manages the forgetting of information, and the output gate $o_{t}$ controls the information output. $h_{t}$ records the output at the current time step, while $c_{t}$ preserves the current cell state and serves as input for the next cell. The sigmoid activation function is denoted by σ, the weight matrix by W, the bias term by b, and the current cell input by $x_{t}$. $s_{t}$ represents the updated state at the current time step, and the hyperbolic tangent activation function is denoted by tanh:


(2) $$\left\{ \begin{array}{l} \begin{array}{c} i_{t}=\sigma \left(W_{i}\left[h_{t-1},x_{t}\right]+b_{i}\right)\\ f_{t}=\sigma \left(W_{f}\left[h_{t-1},x_{t}\right]+b_{f}\right)\\ o_{t}=\sigma \left(W_{f}\left[h_{t-1},x_{t}\right]+b_{o}\right)\\ s_{t}=\tanh\left(W_{s}\left[h_{t-1},x_{t}\right]+b_{s}\right)\\ c_{t}=f_{t}*c_{t-1}+i_{t}*s_{t} \end{array}\\ h_{t}=o_{t}*tanh\left(c_{t}\right) \end{array}\right.$$

BiLSTM consists of a forward LSTM and a backward LSTM, which respectively capture forward and backward information from the text sequence [22]. These are then concatenated to obtain the final hidden layer feature representation. Compared to a single LSTM model, BiLSTM provides a more nuanced understanding of bidirectional semantic dependencies and allows for more precise semantic discrimination [23].

In our model, BiLSTM is applied immediately after the vector representation layer, where ChCancerBERT generates the character-level embeddings tailored to the cancer domain. These embeddings are then fed into the BiLSTM, which consists of a forward and backward LSTMs, capturing both forward and backward contextual information. By leveraging BiLSTM, the model can retain bidirectional semantic dependencies that are crucial in understanding complex medical texts.

### DGCNN Model

DGCNN is a neural network model with residual connections that uses gated dilated convolutions to gather more information through stacked convolutional layers [4]. Its convolutional structure, which enables the model to analyze sequences in segments, examining local patterns that are essential to textual spatial characteristics, which are usually referred to the arrangement and relationships of words and phrases that form meaningful units [4]. These units include nearby dependencies and longer-range relationships, like multiword medical entities in EHRs. DGCNN’s use of dilated convolutions allows it to capture these features at different scales or distances, enabling it to handle both close and distant relationships within the text [14]. The convolution operations proceed as follows:


(3) $$c_{t}=W_{c}\overset{r}{\underset{k=0}{⨁}}x_{t\pm k}$$

In the model, $W_{c}$ denotes a filter with window size *r*, and $c_{t}$ represents the output after convolution transformation. The symbol ⊕ indicates vector concatenation. Building on this, dilation is introduced for interval sampling of data, enabling the coverage of longer sentences. The calculation method for dilated convolution is as follows:


(4) $$c_{t}=W_{c}\overset{r}{\underset{k=0}{⨁}}x_{t\pm k\delta }$$

δ represents the dilation rate, and $W_{c}$ is a filter with window size r. $c_{t}$ denotes the output, while ⊕ signifies vector concatenation. A gating mechanism is also incorporated to control data flow and reduce the risk of gradient vanishing. The operations within each convolutional gated unit are as follows:


(5) $$Y=X+Conv1D_{1}\left(X\right)⊗\sigma \left(Conv2D_{2}\left(X\right)\right)$$

X represents the input. $Conv1D_{1}$ and $Conv2D_{2}$ are dilated convolutions with the same parameter structure but without sharing weights. σ denotes the sigmoid function, and ⊗ represents the Hadamard product of vectors.

After the ChCancerBERT model generates the character-level embeddings, these embeddings are input independently into the DGCNN model, extracting spatial dependencies and local features, which are essential for identifying nested entities and capturing complex structures within cancer-related records.

### MHA Mechanism

The attention mechanism simulates human attention by scoring and weighting important content to highlight significant features [24]. The calculation formula is as follows:


(6) $$Attention\left(Q,K,V\right)=Softmax\left(\frac{QK^{T}}{\sqrt{d_{k}}}\right)V$$

The $Attention\left(Q,K,V\right)$value represents the attention score, where Qs the query vector matrix, K is the key vector matrix, and V is the value vector matrix. $d_{k}$ is the word vector dimension which normalizes the similarity between the query and key vectors calculated via $QK^{T}$ to prevent overly large results. Softmax normalization is then applied, followed by multiplication with the value vector to obtain the final attention value. When vector dimensions are high, a single attention calculation may not capture all features of a word. Thus, the MHA mechanism is used, linearly mapping Q,K,V into multiple subspaces and combining the calculated results:


(7) $$\left\{ \begin{array}{l} head_{i}=Attention\left(QW_{i}^{Q},KW_{i}^{K},VW_{i}^{V}\right)\\ Attention\left(Q,K,V\right)=Softmax\left(\frac{QK^{T}}{\sqrt{d_{k}}}\right)V\\ MultiHead\left(Q,K,V\right)=Concat\left(head_{1},head_{2},\ldots ,head_{k}\right)W^{O} \end{array}\right.$$

This study uses an MHA mechanism to dynamically fuse the BiLSTM and DGCNN models. First, the outputs of both models are mapped to separate feature spaces, generating 2 sets of Q,K,V matrices. Next, the attention values for each model are calculated, resulting in 2 attention representation matrices. Finally, these matrices are combined into a single matrix through matrix multiplication.

### CRF Model

CRF is a sequence modeling framework that incorporates constraints between labels to assign higher probabilities to reasonable label sequences [20]. This approach effectively addresses the issue of label bias.

The probability formula for obtaining the predicted label sequence $X=(x_{1},x_{2},\ldots ,x_{n})$given the input sequence $Y=(y_{1},y_{2},\ldots ,y_{n})$ is as follows:


(8) $$P\left(Y|X\right)=\frac{e^{T\left(X,Y\right)}}{\sum_{Y^{\prime }\in Y_{X}}T\left(X,Y^{\prime }\right)}$$

Given the true label sequence $Y^{`}$, and all possible label sequences $Y_{X}$, the likelihood function for predicting the label sequence Y is as follows:


(9) $$\ln\left(P\left(Y|X\right)\right)=T\left(X,Y\right)-\ln\left(\sum_{Y^{\prime }\in Y_{X}}T\left(X,Y^{\prime }\right)\right)$$

By maximizing the likelihood function, the most probable and reasonable label sequence is determined and output. The specific formula is as follows:


(10) $$Y^{*}=arg_{\tilde{y}\in Y_{X}}maxs\left(X,\tilde{y}\right)$$

### Experiment Setups

#### Dataset Construction

The study’s data were sourced from the inpatient EHRs at a renowned hospital in Beijing, China, spanning from 2013 to 2022. After excluding patients with unrelated admission reasons (ie, those who have previously had breast cancer but whose current reason for admission is not breast cancer), the illness histories from all patients’ admission records within this time period were included. Each patient had 1 record per visit, and we ultimately obtained 876 records of patients with breast cancer (total tokens: 528,925, average length: 659.67, SD 354.23). The data received full approval from the hospital. All procedures in this research adhere to ethical standards. Patient privacy data were deidentified before manipulation, ensuring anonymity and confidentiality of personal information.

#### Annotation Procedure for the Dataset

The annotation of data from 876 admission records of patients with breast cancer was a collaborative effort involving 7 annotators. All of these annotators were the students of the Management School of Beijing University of Chinese Medicine and possessed relevant medical and computer expertise. To enhance the precision of the labeling outcomes, 2 oncology clinicians from the Beijing University of Chinese Medicine were enlisted to train the annotators. They also facilitated content segmentation and offered guidance throughout the labeling process. Based on information extracted from the collected data, in conjunction with insights from pertinent clinical studies [25,26] and guided by categorizations and classifications in clinical literature, as well as referencing indicators present in public medical databases such as SEER, CCKS2017, CCKS2019, along with existing study labeling protocols [27], an index system (Table 2) and labeling criteria (Table 3) were formulated for this research. These encompassed symptoms, tests, treatments, and time.

**Table 2.** Index system of the annotated dataset for Chinese breast cancer electrical health records (EHRs).

Class	Entity
Symptoms	Nodule
Tests	Vascular tumor thrombus and nerve aggress Incisal edge Lymph node dissection Birads TNM^a^ ER^b^ PR^c^ HER2^d^ Ki67
Treatments	Surgery name Chemotherapy Radiotherapy Targeted therapy Endocrine therapy
Time	Time

a TNM: TNM classification of malignant tumors.

b ER: Estrogen receptor.

c PR: progesterone receptor.

d HER2: human epidermal growth factor receptor-2.

**Table 3.** Entity labeling standards with examples^a^.

Entity	Standard	Example	Example in English
Time	Date and time in various formats	2011年	2011
Surgery name	Name of a patient undergoing surgery	左侧乳腺癌根治术	Left breast cancer radical surgery
Nodule	Examination results and location of breast nodules	右乳腺肿物	Right breast mass
Chemotherapy	Patient chemotherapy regimen, medication, cycle	术后行辅助化疗6周期 (紫杉醇+环磷酰胺，具体剂量不详）	After surgery, undergo adjuvant chemotherapy for 6 cycles (paclitaxel+ cyclophosphamide, specific dosages not specified)
Radiotherapy	The patient’s radiotherapy regimen and frequency	放疗25次，具体剂量不详	Undergo 25 sessions of radiation therapy, specific dosage not specified
Targeted therapy	Targeted therapy, drugs	后续贯赫赛汀靶向治疗18次	Followed by 18 sessions of trastuzumab targeted therapy
Endocrine therapy	Endocrine treatment options, drugs	阿那曲唑1mgQd口服内分泌治疗	Anastrozole 1mg once daily, oral administration for endocrine therapy
Incisal edge	Whether the incisal edge of the patient is cancerous	各皮肤切缘未见癌侵润	No cancer infiltration is observed at the margins of the skin
Vascular tumor thrombus and nerve aggression	Results of examination of vascular tumor thrombus and nerve aggression	侵及周围脂肪组织及神经纤维束，脉管中见癌栓	Involvement of surrounding adipose tissue and nerve fiber bundles, with cancer emboli seen in blood vessels
Lymph node dissection	The patient’s lymph node dissection results	淋巴结未见转移癌 (0/21）,腋窝淋巴结 (0/18）, (右腋窝淋巴结）0/3	No metastatic cancer detected in lymph nodes (0/21), axillary lymph nodes (0/18), (right axillary lymph nodes) 0/3
Bi-RADS^b^	Bi-RADS classification of breast nodules	BI_RADS4b类^b^	BI-RADS4b^b^
TNM^c^	The patients’ TNM stages	pT2N0M0, IIa期	pT2N0M0,IIa
ER^d^	Patient pathology reported ER results	ER (+++)	ER (+++)
PR^e^	Patient pathology reported PR results	PR (+++)	PR (+++)
HER2^f^	HER2 results were reported pathologically	HER2 (–)	HER2 (–)
Ki67	Patient pathology reported KI67 results	ki-67index约20%	Ki-67 index is approximately 20%

a All texts to be annotated are in Chinese; for ease of viewing and understanding, the annotated examples in the table are accompanied by corresponding English translations, the patient information presented is simulated.

b Bi-RADS: breast imaging reporting and data system.

c TNM: TNM classification of malignant tumors.

d ER: estrogen receptor.

e PR: progesterone receptor.

f HER2: human epidermal growth factor receptor-2.

The “symptom” labels serve to indicate pathophysiological alterations and breast cancer symptoms, including details about breast nodules such as their location and dimensions. The “tests” label designates the array of tests undergone by patients with breast cancer during diagnosis and treatment, aiding physicians in precise assessments. These encompass parameters like resection margins, vascular thrombosis, nerve invasion, lymph node dissection, Bi-RADS grading, TNM staging, and immunohistochemical markers (ER, PR, HER2, and Ki-67). The “treatments” label encompasses therapies previously administered to patients with breast cancer, comprising chemotherapy, radiation, targeted therapy, endocrine therapy, and surgery. The “time” label incorporates details regarding each temporal point within the admission record of a patient with breast cancer.

Data annotation was performed using Label Studio software (Human Signal). A total of 7 trained annotators, each responsible for a distinct portion of the dataset, performed the initial labeling in strict accordance with the predefined annotation guidelines. The annotated corpus was then independently reviewed and revised by 2 experienced clinical oncologists, resulting in 2 expert-annotated versions. To assess interannotator agreement between the 2 experts, Cohen κ coefficient was calculated, which indicated almost perfect agreement (κ=0.82). Any discrepancies between the 2 expert versions were subsequently identified and resolved through discussion, leading to the final consensus gold-standard dataset. This finalized version was used for training and validation of the entity recognition model.

#### Data Partitioning

For evaluation, we use the hold-out method, partitioning the dataset randomly into training, validation, and test sets, distributed at a ratio of 6:2:2. Diverging from typical NER tasks, this model primarily deals with medical nouns or statements as named entities, with a particular focus on breast cancer–related terms. Table 4 illustrates the count of entities corresponding to each label within the training, validation, and test sets. Concurrently, the length of labels within each category was tabulated, and the outcomes are presented in Table 4.

**Table 4.** Distribution and length statistics of entities in training, validation, and test sets.

Class and entity	Train set, n (%)	Development set, n (%)	Test set, n (%)	Entities length		
				Minimum	Maximum	Mean (SD)
Symptoms						
Nodule	600 (61.66)	192 (19.73)	181 (18.60)	3	123	15.49 (17.84)
Tests						
Vascular tumor thrombus and nerve aggress	208 (56.68)	76 (20.71)	83 (22.62)	4	42	13.93 (7.79)
Incisal edge	155 (58.49)	53 (20)	57 (21.51)	4	75	24.15 (17.06)
Lymph node dissection	558 (62.21)	137 (15.27)	202 (22.52)	4	139	27.92 (24.67)
Bi-RADS^a^	161 (54.39)	58 (19.59)	77 (26.01)	8	82	48.59 (22.54)
TNM^b^	55 (57.89)	15 (15.79)	25 (26.32)	5	17	10.97 (3.76)
ER^c^	438 (61.78)	132 (18.62)	139 (19.61)	3	45	9.7 (5.52)
PR^d^	430 (61.69)	131 (18.79)	136 (19.51)	2	33	5.61 (4.88)
HER2^e^	393 (61.50)	117 (18.31)	129 (20.19)	5	82	13.64 (10.36)
Ki67	420 (62.22)	132 (19.56)	123 (18.22)	7	59	14.66 (7.06)
Treatments						
Surgery name	985 (60.39)	304 (18.64)	342 (20.97)	3	74	18.91 (9.06)
Chemotherapy	1027 (61.72)	319 (19.17)	318 (19.11)	2	940	101.43 (107.68)
Radiotherapy	177 (62.32)	56 (19.72)	51 (17.96)	2	141	21.32 (21.42)
Targeted therapy	155 (65.96)	30 (12.77)	50 (21.28)	5	352	68.83 (68.16)
Endocrine therapy	229 (61.89)	71 (19.19)	70 (18.92)	5	202	32.55 (26.9)
Time						
Time	2765 (59.54)	892 (19.21)	987 (21.25)	2	395	56.41 (55.25)

a Bi-RADS: breast imaging reporting and data system.

b TNM: TNM classification of malignant tumors.

c ER: estrogen receptor.

d PR: progesterone receptor.

e HER2: human epidermal growth factor receptor-2.

#### Evaluation Metrics

During the experiment, the evaluation of the NER model used 3 metrics: precision (P), recall (R), and *F*_1_*-*score (*F*_1_). The formulas for calculating precision, recall, and *F*_1_-score are as follows:


(11) $$\left\{ \begin{array}{l} P=\frac{TP}{TP+FP}\times 100\%\\ R=\frac{TP}{TP+FN}\times 100\%\\ F1=\frac{2PR}{P+R}\times 100\% \end{array}\right.$$

where TP represents the number of true positive entities, FP indicates the number of false positive entities, and FN denotes the number of false negative entities.

#### Implementation Details

The experiments were conducted using the PyTorch 1.6 framework in a Python (version 3.7; Python Software Foundation) environment. The CPU used is Intel(R) Core i5-12500H, and the GPU used is NVIDIA T4 GPU. The ChCancerBERT model parameters were configured identically to the BERT model, with 12 transformer layers, 12 attention heads, and 768 hidden units. The hyperparameters for the NER experiment are detailed in Table 5.

**Table 5.** Hyperparameter settings.

Hyperparameters	Value
Batch size	8
Dropout	0.5
Learning rate	0.00003
Epoch	64
Maximum length	128
Embedding	768

## Results

Using the partitioned dataset, we devised comparative experiments, ablation experiments, and separate pretrained model comparative experiments to facilitate a comprehensive assessment of the recognition efficacy across various mechanisms and models.

### Results of the Comparative Experiment for NER Models

We applied our proposed model and the NER models mentioned in Table 1, which have been developed for medical and cancer fields in recent years, to our dataset to compare performance. Detailed results can be found in Table 6. We did not include Zhou et al CancerBERT model [7] in the comparison because it is not yet publicly available. Our results demonstrate that, for the Chinese breast cancer EHR dataset we constructed, our proposed model outperformed existing models, with *F*_1_-scores increasing by 1.40%, 2.57%, and 1.13%, respectively.

**Table 6.** Comparative performance of baseline and proposed models on the breast cancer dataset.

Model	Precision (%)	Recall (%)	*F*_1_-score (%)
BERT^a^- BiLSTM^b^ -CRF [5]	85.77	85.38	85.58
ALBERT^c^-IDCNN^d^-MHA-CRF [15]	87.78	81.28	84.41
MC-BERT-BiLSTM-CNN^e^-MHA^f^-CRF^g^ [6]	85.64	86.07	85.85
Our proposed model	87.63	86.34	86.98

a BERT: Bidirectional Encoder Representations from Transformers.

b BiLSTM: bidirectional long short-term memory.

c ALBERT: A Lite Bidirectional Encoder Representations from Transformers.

d IDCNN: iterated dilated convolutional neural network.

e CNN: convolutional neural network.

f MHA: multihead attention.

g CRF: conditional random field.

### Results of the Ablation Experiment

We employ the fundamental BERT-BiLSTM-CRF model as a baseline to assess the impact of distinct mechanisms on the recognition of named entities within Chinese EHRs pertaining to breast cancer. Subsequently, BERT-DGCNN-CRF replaces the BiLSTM model within the baseline, while the baseline+DGCNN model merges the BiLSTM and DGCNN models. In addition, the baseline+DGCNN+MHA model introduces a MHA mechanism to the BERT-BiLSTM-DGCNN-CRF foundation. The corresponding experimental outcomes are detailed in Table 7.

Analysis of the ablation outcomes reveals that the substitution of the BiLSTM model with the DGCNN model results in a reduction of the precision and *F*_1_*-*score on the dataset of this study by 0.37% and 0.14%. However, the amalgamation of the DGCNN model and the BILSTM model yields *F*_1_*-*score increments of 0.35% and 0.49% compared to both the baseline and BERT-DGCNN-CRF models. This amalgamation also improves precision by 0.02% and 0.29%, and recall by 0.69%, compared to the baseline and BERT-DGCNN-CRF models, respectively. This observation underscores that, within the context of feature extraction, the standalone DGCNN model presents no notable superiority over the BiLSTM model. Conversely, the combined application of these 2 models augments recognition effectiveness. Building upon this foundation, the incorporation of the MHA serves to enhance the extraction of pertinent information within the data, thereby mitigating the impact of irrelevant data. Consequently, the *F*_1_*-*score experiences a supplementary enhancement of 0.17%, with a precision decrease of 0.46% offset by a substantial recall increase of 0.82%. This suggests that although the addition of the MHA slightly reduces precision, it compensates for this by capturing a broader range of relevant information, ultimately enhancing overall recognition effectiveness. Thus, based on the insights garnered from ablation experiments, it is ascertained that the optimal approach for this study involves the incorporation of the BiLSTM-DGCNN fusion model as the feature extraction layer, accompanied by the integration of the MHA mechanism.

**Table 7.** Results of ablation studies and pretrained model comparisons.

Model	Precision (%)	Recall (%)	*F*_1_-score (%)
BERT^a^-BiLSTM^b^-CRF^c^ (baseline)	85.77	85.38	85.58
BERT-DGCNN^d^-CRF	85.50	85.38	85.44
BERT-DGCNN-BiLSTM-CRF	85.79	86.07	85.93
BERT-DGCNN-BiLSTM-MHA^e^-CRF	85.33	86.89	86.10
ALBERT^f^-BiLSTM-CRF	87.50	77.32	82.10
ALBERT-DGCNN-BiLSTM-MHA-CRF	86.61	84.98	85.78
RoBERTa^g^-BiLSTM-CRF	86.14	82.51	84.29
RoBERTa-DGCNN-BiLSTM-MHA-CRF	86.86	85.79	86.32
XLNet^h^-BiLSTM-CRF	86.66	78.55	82.40
XLNet-DGCNN-BiLSTM-MHA-CRF	87.52	85.93	86.72
MC-BERT-BiLSTM-CRF	85.36	85.25	85.30
MC-BERT-DGCNN-BiLSTM-MHA-CRF	86.98	86.48	86.73
ChCancerBERT-BiLSTM-CRF	85.75	86.61	86.18
Our proposed model	87.63	86.34	86.98

a BERT: Bidirectional Encoder Representations from Transformers.

b BiLSTM: bidirectional long short-term memory.

c CRF: conditional random field.

d DGCNN: dilated gated convolutional neural network.

e MHA: multihead attention.

f ALBERT: A Lite Bidirectional Encoder Representations from Transformers.

g RoBERTa: robustly optimized Bidirectional Encoder Representations from Transformers pretraining approach.

h XLNet: generalized autoregressive pretraining.

### Results of the Comparative Experiment for the Pretrained Models

Leveraging the model exhibiting the highest *F*_1_*-*score from the aforementioned experiments, we proceeded to perform comparative experiments by substituting various pretrained models. Comparison experiments were conducted by integrating different pretrained models with both BiLSTM+CRF and DGCNN-BiLSTM-MHA-CRF architectures. All used pretrained models are available for download from the “Models-Hugging Face” website. The corresponding outcomes are presented in Table 7.

The results indicate that using the proposed pretraining model, ChCancerBERT, for character-level embedding outperforms various pretrained models compared in the experiment. Specifically, in the comparison experiment combined with BiLSTM+CRF, the *F*_1_*-*scores of the ChCancerBERT model increased by 0.6%, 4.08%, 1.89%, 3.78%, and 0.88% compared to BERT, ALBERT, RoBERTa, XLNet, and MC-BERT, respectively. In the comparison experiment with the DGCNN-BiLSTM-MHA-CRF architecture, it achieved *F*_1_*-*score improvements of 0.88%, 1.20%, 0.66%, 0.26%, and 0.25% over BERT, ALBERT, RoBERTa, XLNet, and MC-BERT, respectively. In addition, regardless of the pretraining model used, the *F*_1_*-*scores obtained with the DGCNN-BiLSTM-MHA-CRF architecture were consistently higher than those obtained with the BiLSTM+CRF model alone, further demonstrating that the DGCNN-BiLSTM-MHA model, by integrating multidimensional text features, possesses a stronger semantic representation capability than the BiLSTM model alone.

After using ChCancerBERT for character-level embedding and integrating it with the DGCNN-BILSTM-MHA-CRF model to fuse multidimensional text features, the semantic representation capability is further enhanced, and the *F*_1_*-*score had a 1.4% improvement compared to the current mainstream BERT-BiLSTM-CRF (baseline) model. In addition, our model achieves a precision of 87.63% and a recall of 86.34%, which represents increases of 1.86% in precision and 0.96% in recall compared to the baseline BERT-BiLSTM-CRF model. This reflects both high detection accuracy and robustness in capturing target entities.

### Error Analysis

We conducted a comprehensive analysis of the 4 main entity categories in this study, including their quantity, average length, as well as precision, recall, and *F*_1_*-*scores. The specific results are presented in Table 8.

The results presented in Table 8 indicate that TIME entities are the most frequently occurring, totaling 4644 instances. The model demonstrates the highest recognition effectiveness for these entities, achieving an *F*_1_*-*score of 92.79%. In contrast, although the treatments category also has a high number of entities, it shows the lowest recognition performance with an *F*_1_*-*score of 74.24%. This lower performance can be attributed to the complexity and variability of treatment-related terms, which is further compounded by the fact that the average length of treatment entities is 17.27 characters, significantly longer than other entity categories. Treatment entities often contain multiple details, including various drugs, dosages, treatment methods, and frequencies, such as in the example: “Chemotherapy: Cyclophosphamide 1000 mg on day 1, Epirubicin 50 mg on day 1, 21 days per cycle, 4 cycles.” However, the model only identifies the drugs and their dosages used in chemotherapy, while ignoring the subsequent frequency information. The increased length and complexity of these entities make it more challenging for the model to accurately identify and classify them, resulting in a decrease in overall recognition accuracy.

**Table 8.** Performance obtained for each entity.

Class	Entity number	Average length	Precision (%)	Recall (%)	*F*_1_*-*score (%)
Symptoms	973	7.62	82.86	84.89	83.86
Tests	640	7.82	82.61	81.40	81.02
Treatments	4184	17.27	76.90	72.30	74.24
Time	4644	8.01	92.62	92.96	92.79

### Experiment on CCKS2019

In order to comprehensively validate the efficacy of the model proposed in this study, we applied it to the CCKS2019 dataset and conducted a performance comparison with models recently introduced in the literature. The CCKS2019 dataset, publicly accessible and broadly used within China’s medical NLP research community, offers a standardized evaluation platform for diverse NER models (CCKS2019 [28]). In total, we compared 8 models, including 3 models in 5.1, and the other 5 models are as follows:

- Embeddings from language models (ELMo)-lattice-LSTM-CRF model: this model uses the lattice LSTM-CRF architecture and integrates variant contextual character representations (Chinese Embeddings from Language Models [ELMo]) to enhance the use of character and word information in Chinese EHRs [29].
- ELMo-encoder from transformer–CRF model: this model fine-tunes the ELMo model using domain-specific clinical records and uses transformer (encoder from transformer) as an encoder to address long-context dependencies [30].
- All CNN model: this model incorporates Chinese radical characters to enhance the morphosemantic representation of Chinese characters [14].
- Graph attention network–BiLSTM-CRF model: this model uses a graph neural network to model lexical dependency inertia as syntactic semantics and integrates semantic information at various levels to enhance contextual representation [31].
- Chinese medical–NER model: this model enhances the MC-BERT model by incorporating graph convolutional networks and CRFs [16].

Table 9 presents a comparison of the performance of our proposed model on the CCKS2019 dataset with that of models introduced in recent literature. Our model achieves an *F*_1_*-*score of 87.26%, surpassing recent models by margins of 2.64%, 2.24%, 2.13%, 1.67%, 1.63%, 1.34%, 0.99%, and 0.97%. These results strongly affirm the effectiveness of our model. The experimental findings clearly demonstrate that our cancer-specific named NER model outperforms existing models on the CCKS2019 dataset, highlighting its potential for effectively handling larger medical datasets.

**Table 9.** Performance comparison of the proposed model and recent models on the CCKS2019 dataset.

Model	Precision (%)	Recall (%)	*F*_1_*-*score (%)
BERT^a^- BiLSTM^b^ -CRF^c^ [5]	82.09	87.32	84.62
ELMo^d^-lattice-LSTM^e^-CRF [29]	85.35	84.69	85.02
ACNN^f^ [14]	87.29	83.07	85.13
ELMo-ET^g^-CRF model [30]	87.61	83.65	85.59
ALBERT^h^-IDCNN^i^-MHA^j^-CRF [15]	84.82	86.46	85.63
GAT^k^-BiLSTM-CRF [31]	86.74	85.11	85.92
MC-BERT-BiLSTM-CNN^l^-MHA-CRF [6]	84.90	87.67	86.27
CM^m^-NER^n^ [16]	86.45	86.13	86.29
Our proposed model	87.26	87.27	87.26

a BERT: Bidirectional Encoder Representations from Transformers.

b BiLSTM: bidirectional long short-term memory.

c CRF: conditional random field.

d ELMo: embeddings from language models.

e LSTM: long short-term memory.

f ACNN: all convolutional neural network.

g ET: encoder from transformer.

h ALBERT: A Lite Bidirectional Encoder Representations from Transformers.

i IDCNN: iterated dilated convolutional neural network.

j MHA: multihead attention.

k GAT: graph attention network.

l CNN: convolutional neural network.

m CM: Chinese medical.

n NER: named entity recognition.

## Discussion

### Principal Findings

Unstructured EHR data contains valuable patient information that can be used for clinical decision support and research [32]. In the Chinese breast cancer EHR corpus constructed in this study, there are 876 admission records, comprising a total of 528,925 tokens. Annotations were performed for 4 major categories and 17 subcategories of named entities, with each subcategory having more than 100 annotated instances. In the NER task, the model we propose outperforms other models currently applied in medical-related fields. The results of ablation experiments revealed that the fusion of the pretrained model with the DGCNN-BILSTM-MHA-CRF model effectively enhances entity recognition performance. Specifically, the introduction of the DGCNN structure on top of the baseline results in the maximum improvement of 0.35%. This enhancement is attributed to the structure’s ability to mitigate the risk of gradient vanishing and facilitate information transmission across multiple channels, capturing a wealth of feature information. In addition, the incorporation of the MHA mechanism on this foundation not only enhances the model’s generalization capability but also enables parallel attention computation, strengthening feature fusion capability [24]. This leads to improved use of valid information and reduced impact of irrelevant information, resulting in a further *F*_1_*-*score improvement of 0.17%.

Comparing the results of the pretrained model experiments, it is evident that the proposed ChCancerBERT pretrained model outperforms various state-of-the-art BERT models, with the medical domain-specific MC-BERT model ranking second. This is attributed to MC-BERT incorporating more medical corpus training into BERT, and ChCancerBERT further including the Chinese cancer corpus on top of MC-BERT. This augmentation effectively enhances the model’s semantic representation capability in the Chinese cancer domain, yielding superior results in the Chinese breast cancer corpus in this study. In addition, the study’s results indicate that pretrained BERT models based on specific domains can lead to improved performance in downstream tasks [7].

Among the 4 categories extracted in this study, the performance in extracting time entities stood out, with an impressive *F*_1_*-*score of 92.79%. This result can be attributed to the relatively high quantity of time entities in this dataset, amounting to 4644, and the fact that time entities exhibit a more standardized writing format compared to other entities, which, to some extent, reduces the difficulty of recognition and enhances recognition accuracy [33].

We scrutinize the entity recognition outcomes of the most proficient fusion model derived from our experiments. Categories with infrequent occurrences in the dataset, such as TNM, Bi-RADS, and some other entities in class “tests,” are prone to omission errors. These errors manifest as instances where entities that should have been identified remain undetected, suggesting a propensity of the model used in this study to overlook less common entity categories [34]. Such omissions may result in incomplete information extraction, which is crucial for disease diagnosis and the formulation of treatment plans. Therefore, downstream applications should incorporate clinician verification mechanisms or error correction modules to ensure safety in clinical use.

In this study, entities characterized by considerable length, such as chemotherapy, radiotherapy, and other in-class “treatments,” are typically classified correctly by the model. However, the model is susceptible to boundary recognition errors [7], leading to either the exclusion of words at the sentence’s periphery or the inclusion of extraneous words beyond the sentence, which may cause confusion in clinical documentation. Furthermore, on one hand, Chinese words frequently exhibit polysemy and ambiguity [33]; on the other hand, the dataset for this study contains instances where statements are nested within the entity categories of other entities. Such intricacies heighten the risk of erroneous entity category determinations.

Concurrently, we apply the model introduced in this research to the publicly accessible CCKS2019 dataset, and the findings illustrate its superior performance compared to prior research efforts, suggesting the ChCancerBERT framework has the potential to be extended to other cancer types and even broader clinical domains. Nevertheless, differences in documentation style and terminology across hospitals may affect generalization. Domain adaptation strategies such as continual pretraining on local EHR corpora and transfer learning can be explored to improve portability to other institutions.

Using our proposed model, vital clinical data encompassing timestamps, examination details, symptoms, and treatment modalities can be readily extracted from the EHRs of patients with cancer. The extracted entities provide important value for both clinical and research applications. Time, symptom, test, and treatment information can be structured into patient timelines to support disease monitoring, clinical decision support, population health analysis, and trial recruitment. These entities can also be mapped to standards such as International Classification of Diseases, 10th Revision, enabling interoperability and secondary use of structured cancer data.

### Conclusions

This study designed and developed a pretrained model specifically tailored to the Chinese cancer domain, named ChCancerBERT. The model was then integrated with various NER models proposed in the field of computer science, resulting in the ChCancerBERT-DGCNN-BILSTM-MHA-CRF model. This integrated model was used to extract 4 major types of entities related to cancer from admission records in the oncology department of hospitals. We applied the model proposed in this study to Chinese breast cancer EHR data and CCKS2019 for experimentation and validation. The proposed model achieved an *F*_1_*-*score of 86.98%, surpassing other models compared in the experiment.

The primary limitation of this study is the restricted data accessibility and relatively small sample size. This limited sample size may affect the statistical power and robustness of the findings. Moreover, potential variations in clinical documentation practices across different regions or institutions in China could pose challenges to the generalizability of the model. Despite these limitations, it is worth noting that the model demonstrated promising transferable capability by achieving competitive performance on the CCKS2019 medical dataset, which suggests its potential applicability beyond breast cancer data. Therefore, future research should aim to acquire EHR data for other types of cancer to further validate the generalizability of the proposed model. Furthermore, a NER system tailored for clinical application is envisaged to be constructed based on this model. This system aims to assist clinical researchers in data organization and extraction. In addition, plans encompass the establishment of a clinical decision support system to facilitate more effective treatment for patients.

## Abbreviations

BiLSTM: bidirectional long short-term memory.
DGCNN: dilated-gated convolutional neural network
EHR: electronic health record
ELMo: embeddings from language models
LSTM: long short-term memory
MHA: multihead attention
NER: named entity recognition
NLP: natural language processing
